# Porcine Circoviruses and Herpesviruses Are Prevalent in an Austrian Game Population

**DOI:** 10.3390/pathogens11030305

**Published:** 2022-02-28

**Authors:** Angelika Auer, Lea Schweitzer, Anna Kübber-Heiss, Annika Posautz, Katharina Dimmel, Kerstin Seitz, Christoph Beiglböck, Christiane Riedel, Till Rümenapf

**Affiliations:** 1 Institute of Virology, Department of Pathobiology, University of Veterinary Medicine, 1210 Vienna, Austria; angelika.auer@vetmeduni.ac.at (A.A.); 11774488@students.vetmeduni.ac.at (L.S.); katharina.dimmel@vetmeduni.ac.at (K.D.); kerstin.seitz@vetmeduni.ac.at (K.S.); till.ruemenapf@vetmeduni.ac.at (T.R.); 2 Research Institute of Wildlife Ecology (FIWI), Department of Interdisciplinary Life Sciences, University of Veterinary Medicine, 1160 Vienna, Austria; anna.kuebber@vetmeduni.ac.at (A.K.-H.); annika.posautz@vetmeduni.ac.at (A.P.); christoph.beiglboeck@vetmeduni.ac.at (C.B.)

**Keywords:** wild boar, wild ruminants, porcine lymphotropic herpesvirus, PCV2, PCV3, ruminant gammaherpesvirus

## Abstract

During the annual hunt in a privately owned Austrian game population in fall 2019 and 2020, 64 red deer (*Cervus elaphus*), 5 fallow deer (*Dama dama*), 6 mouflon (*Ovis gmelini musimon*), and 95 wild boars (*Sus scrofa*) were shot and sampled for PCR testing. Pools of spleen, lung, and tonsillar swabs were screened for specific nucleic acids of porcine circoviruses. Wild ruminants were additionally tested for herpesviruses and pestiviruses, and wild boars were screened for pseudorabies virus (PrV) and porcine lymphotropic herpesviruses (PLHV-1-3). PCV2 was detectable in 5% (3 of 64) of red deer and 75% (71 of 95) of wild boar samples. In addition, 24 wild boar samples (25%) but none of the ruminants tested positive for PCV3 specific nucleic acids. Herpesviruses were detected in 15 (20%) ruminant samples. Sequence analyses showed the closest relationships to fallow deer herpesvirus and elk gammaherpesvirus. In wild boars, PLHV-1 was detectable in 10 (11%), PLHV-2 in 44 (46%), and PLHV-3 in 66 (69%) of animals, including 36 double and 3 triple infections. No pestiviruses were detectable in any ruminant samples, and all wild boar samples were negative in PrV-PCR. Our data demonstrate a high prevalence of PCV2 and PLHVs in an Austrian game population, confirm the presence of PCV3 in Austrian wild boars, and indicate a low risk of spillover of notifiable animal diseases into the domestic animal population.

## 1. Introduction

Wild animal populations can be affected by diseases introduced by domestic animals, but can also be conceived as a threat to domestic animal populations as potential vectors or reservoirs of disease. Therefore, monitoring wildlife health is an important tool to analyze disease dynamics in wild populations and the effect and likelihoods of spill-over to domestic animals and vice versa.

Important pathogens of domestic animals that are also present in wild ruminants and wild boar are, for example circoviruses, herpesviruses, and pestiviruses. Porcine circovirus type 2 (PCV2) is a small, circular, single-stranded DNA virus of the family *Circoviridae*. It plays an important role in pig production worldwide and is known to cause postweaning multisystemic wasting syndrome (PMWS); porcine dermatitis and nephropathy syndrome (PDNS); and other diseases such as respiratory problems, enteritis, and reproductive failure [1,2,3,4,5]. PCV2 is highly prevalent in European wild boar populations, but associated clinical disease is rarely observed [6,7,8,9,10,11,12,13,14,15].

Porcine circovirus type 3 (PCV3) was discovered in pigs in 2016 and is highly prevalent in the domestic pig population [16]. It is associated with different syndromes, such as PDNS and reproductive failure [17,18,19,20,21,22,23,24,25,26,27,28,29,30,31,32,33,34]. Comparable to PCV2, PCV3 can also be detected in healthy pigs, and its distribution in the wild boar population seems to be similar to domestic pigs, ranging from 23% to 50% [15,35]. Porcine circoviruses were also detected in chamois, roe deer, dogs, cattle, mice, and ticks, but the clinical relevance in these species is unclear [36,37,38,39,40,41].

Aujeszky’s disease is caused by suid alphaherpesvirus 1 (SuHV-1, PrV) and is endemic in most parts of the world. Its economic impact led to its eradication from the domestic pig populations of many European countries. PrV remains prevalent in European wild boar populations [42,43,44,45] and mostly results in asymptomatic infection. It causes rabies-like and always fatal disease in many different mammalian species other than swine. Hunting dogs, which come into contact with blood or raw wild boar meat, are frequently infected [46,47].

The role of gammaherpesviruses in human and animal health is still not fully understood. As they have certain sequence homologies to human herpesviruses (HHV-4/EBV and HHV-8), porcine lymphotropic herpesviruses (PLHV-1, -2, and -3) has raised the interest of scientists investigating pig-to-human xenotransplantation and post-transplant lymphoproliferative disease (PLTD). PLHV-1, -2, and -3 are highly prevalent in domestic pigs in Germany, Italy, Ireland, and Spain. Infected animals are clinically healthy [48,49,50,51], but an influence on the host’s immune system caused by the infection of B-lymphocytes has been proposed by the authors of [49]. PLHV-1 and -2 were also detected in a German feral pig population, but only a few samples have been analyzed to date [48,49].

Gammaherpesviruses of the *Lymphocryptovirus* genus, such as ovine herpesvirus 2 (OvHV-2), caprine herpesvirus 2 (CpHV-2), and alcephaline herpesvirus 1 (AlHV-1), are well adapted to their host’s immune system and mainly cause subclinical infections. They can cause lymphoproliferative malignant catarrhal fever (MCF) if transmitted to cattle, buffalo, bison, sika deer, or even pigs [52,53,54,55,56,57]. Other gammaherpesviruses like elk gammaherpesvirus or fallow deer herpesvirus are not known to be associated with disease. They may influence the animal´s immune system or can cause lymphoproliferative diseases in other species [58,59].

The ruminant pestiviruses border disease virus (BDV) and bovine viral diarrhea virus (BVDV) (family *Flaviviridae*) can infect various ungulate species. Starting in 2004, a national eradication program succeeded to eliminate BVDV from Austrian cattle herds. Still, there is a certain risk of re-introduction of pestiviruses into cattle herds by indirect contact with infected deer. Reported seroprevalences in Austria and Switzerland are low (~2%). In Spain, seroprevalences range from 11% to 20% in red deer samples, and it has been demonstrated that wild ruminants have the potential to transmit the virus [60,61,62,63]

In the present study, we generated a snapshot of the circulation of circo-, herpes-, and pesti-viruses, as well as the morbidities present at necropsy in an Austrian game population. The study’s aim is to increase our understanding of the circulation and viral loads of relevant pathogens of domestic animals in Austrian game, and to further our knowledge of the biology of gammaherpesviruses and their pathological significance in game species. Here, 75 wild ruminants and 95 wild boars shot during canned hunts in fall 2019 and 2020 were sampled for the purpose of this study. Different herpes virus species were identified by sequence analysis in the ruminant population, and the viral loads of PCV2; PCV3; and PLHV-1, -2, and -3 were analyzed. The studied game population lives in a 12 km^2^ forest area surrounded by a stone wall and has not been restocked for at least ten years.

## 2. Results

Most animals in this study had a moderate to good body condition (BCS 2 or larger; wild boar 95% and wild ruminants 87%). Poor body condition was only observed in juvenile animals. The gross pathological examination revealed enteritis in 30% of the wild ruminants, lung lesions in 17%, liver lesions in 21%, and localized lymphadenopathy in 39% of the wild ruminants. For wild boar, milk spots (chronic interstitial hepatitis caused by *Ascaris suum*) were detected in 23%, intestinal parasites in 85%, and lungworms in 46% of the individuals sampled. In addition, 18% of the wild boar exhibited localized lymphadenopathy and 7% exhibited a suppurative lymphadenitis or abscesses in the mandibular lymphnodes.

During necropsy, samples were taken by aspiration with sterile needles from the liver and spleen to avoid environmental contamination and cross-contamination between different animals. Viral nucleic acids extracted from tissue samples and swabs were analyzed for the presence of the following viral pathogens: wild ruminants, herpesviruses, pestiviruses, PCV2, and PCV3, and wild boar, PCV2 and 3, PLHV-1−3, and PrV. The presence of viruses and virus infection were analyzed for associations with age, BCS, and comorbidities.

**Figure 1:**
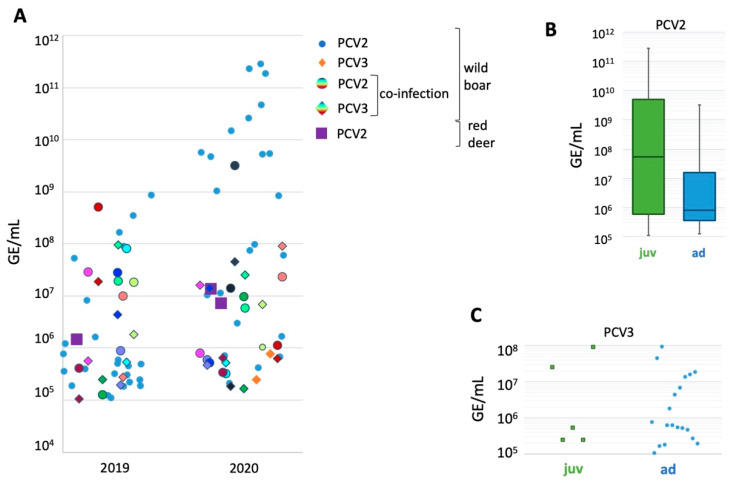
Virus loads of PCV2 and PCV3 in wild boar and red deer samples in GE (genome equivalents)/mL sample. (**A**) Samples of wild boar only testing positive for PCV2 are depicted as blue dots, and those testing only positive for PCV3 as orange diamonds. Values of wild boar samples testing positive for PCV2 (dot) and PCV3 (diamond) are indicated by the same color for each individual sample. Red deer samples positive for PCV2 are indicated by purple squares. No PCV3 infection was detected in wild ruminants. PCV2 (**B**) and PCV3 (**C**) virus loads depending on the age of the sampled wild boar.

In 34 out of 50 (68%) wild boar samples in 2019 and 37 out of 45 in 2020 (82%), PCV2 was detected by qPCR (Figure 1).

Viral loads ranged from 1.12 × 10^5^ GE (genome equivalents)/mL to 8.75 × 10^8^ GE/mL in 2019 (average 6.63 × 10^7^, viral loads are given in GE/mL due to the sampling strategy used). In 2020, 2.1 × 10^5^ to 2.9 × 10^11^ GE/mL (average 2.2 × 10^10^) were present in the samples of positive wild boars, and 10 wild boars had viral loads above 1 × 10^9^ GE/mL sample. The PCV2 viral loads in 2019 and 2020 differed significantly (*p* = 0.048). Higher virus loads were significantly associated with the age of the animals (*p* = 0.049), with juveniles presenting with higher loads (Figure 1B).

In 2019, 10 wild boars positive for PCV2 also tested positive for PCV3 (29% of PCV2 positive animals, 20% of animals tested, GE/mL ranging from 1.1 × 10^5^ to 9.5 × 10^7^). Two wild boars sampled in 2020 only tested positive for PCV3 (4.4%), while 12 out of 37 PCV2 positive wild boars also were infected with PCV3 (32% of PCV2 positive individuals, 27% of individuals tested, GE/mL ranging from 1.7 × 10^5^ to 9.1 × 10^7^). At least in this small study population, PCV3 seemed to be predominantly present as co-infection with PCV2. PCV3 infection was significantly associated with lower PCV2 loads in 2020 (*p* = 0.049). No PCV3 infection was observed in wild boars with a BCS below 2 (all data generated by this study are available in Supplementary Table S1) and the infection was significantly associated with age (11% of juveniles virus positive and 37% of adults virus positive, *p* < 0.01), while the virus loads seemed comparable (Figure 1C).

Three out of 75 wild ruminants (4%) tested positive for PCV2 specific nucleic acids with virus loads of 1.46 × 10^6^, 1.35 × 10^7^, and 7.25 × 10^6^ GE/mL sample. These three individuals were in good body condition, and both age groups and sexes were represented. PCV3 could not be detected in any wild ruminant.

**Figure 2:**
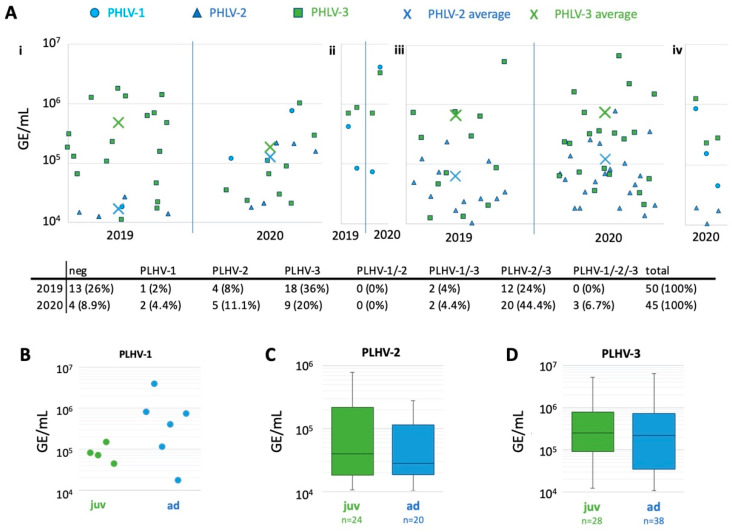
Virus loads of PLHVs in wild boar samples in 2019 and 2020. (**A**) PLHV-1 is indicated by cyan dots, PLHV-2 by blue triangles, PLHV-3 by green squares, and the averages by crosses. If several viruses were detected in the same sample, the values for the different PLHV species are given on the same location of the X-axis. (**i**) Viral loads in wild boar infected with one PLHV. (**ii**) Viral loads in wild boar infected with PLHV-1 and -3. (**iii**) Viral loads in wild boar infected with PLHV-2 and -3. (**iv**) Viral loads in wild boar infected with all three PLHVs. The number of animals belonging to each category is given in the table below the graphs. (**B**–**D**) Virus loads of PLHVs depending on the age of the sampled wild boar.

Porcine lymphotropic herpesviruses (PLHV) were frequently detected in the wild boar samples (74% individuals tested positive in 2019 and 91.1% in 2020) (Figure 2), as single, double, or triple infections.

The dominant species was PLHV-3. It was present in 32 samples in 2019 (64%) and 34 (samples) in 2020 (76%). PLHV-2 was detected in 16 samples in 2019 (32%) and 28 samples in 2020 (62.2%). PLHV-1 was least represented in the population, with three positive wild boars in 2019 (6%) and seven positive wild boars in 2020 (16%). The predominant PLHV infection was co-infection of PLHV-2 and -3, which was detected in 32 wild boars during the study period (34%). Interestingly, no co-infection of PLHV-1 and -2 was observed, while three triple infections with PLHV-1, -2, and -3 were detected in 2020 (6.7%). Viral loads in GE/mL sample ranged from 1.8 × 10^4^ to 4.0 × 10^6^ for PLHV-1 (average 6.4 × 10^5^), from 1.0 × 10^4^ to 7.8 × 10^5^ for PLHV-2 (average 9.0 × 10^4^), and from 1.1 × 10^4^ to 5.1 × 10^6^ for PLHV-3 (average 6.1 × 10^5^). No statistically significant association of GE levels and co-infection was observed. PLHV viral loads did not significantly differ between juvenile and adult animals (Figure 2B–D), but PLHV-1 loads were generally lower in juvenile animals.

It is still unclear whether PLHV infections are associated with additional comorbidities. To gather further evidence, we analyzed the virus loads of PCV2, PCV3, and PLHV-1 to -3, taking signs of parasite burden—defined as the presence of intestinal parasites, milk spots, and lung worms—as observed during necropsy into account. A significant association between viral loads and lesions found at necropsy was observed in two cases (Supplementary Figure S1). Lower viral loads of PCV2 and PLHV-3 were observed when intestinal parasites were absent.

Herpesviruses were detected in 12 out of 75 ruminant samples (16%; four animals in 2019 and eight animals in 2020) by PCR (Table 1). Sequence analysis of the PCR products revealed the closest similarity to fallow deer herpesvirus in ten cases (two samples derived from fallow deer and eight derived from red deer), and two sequences (162 bp) derived from red deer were closest related (99.4%) to elk gammaherpesvirus (GenBank: KY612411.1.)

## 3. Discussion

In the present study, 170 game animals living in a 12 km^2^ game reserve shot during canned hunts in 2019 and 2020 were sampled and screened for different viruses. High-quality sampling of such large numbers of animals in a time- and cost-efficient manner remains challenging. To accelerate this process and avoid cross-contamination and the need for large numbers of sterile and DNA-free knives, scissors, and tweezers, tissue samples were aspirated using sterile syringes and large-bore needles. Subsequently, the tissue material could be processed under sterile laboratory conditions. We consider this way of sampling tissue of shot animals very practical and time effective, while the risk of cross-contamination is minimized.

Monitoring animal populations for known and novel pathogens is a key aspect to assess the risk—in the case of known animal pathogens—of transmission to domestic animals and spill-over events. Additionally, it is central to monitor the health status of a population and provides novel insights into disease dynamics. In this study, the presence of several pathogens was for the first time analyzed in a quantitative manner in an Austrian game population.

According to known literature, PCV2 prevalence in wild boar populations shows high variations in different regions. Hammer et al. (2012) found feral pig populations of three woodland areas in Baden-Wurtemburg (Germany) to be 17%, 37%, and 98% positive for PCV2 specific nucleic acids. In a study by Reiner et al. (2010) [11], 522 wild boar samples from 14 German districts showed a mean qPCR positivity of 45%. In the population studied here, 74% of wild boar samples tested positive for PCV2, placing our population towards the upper end of the reported prevalence in central Europe. We determined mean viral loads of 1.15 × 10^10^ GE/mL tissue and swab lysate, which is high compared to Hammer et al. (2012) [13], who published mean viral loads of ~2.51 × 10^8^ GE/mL tissue lysate. Reiner et al. (2010) [11] reported 10^2.8^ GE/µg DNA on average. The significant difference in PCV2 viral loads in our study population between the two sampling events was likely caused by the high PCV2 viral loads in juvenile animals in 2020, and indicates an acute outbreak of PCV2, which would increase the likelihood of clinical disease [64]. However, no increase in overtly diseased animals was observed by the hunters surveilling the resort (personal communication), indicating that high viral loads are less likely to be associated with overt disease in wild boars than in domestic pigs. More data, including detailed patho-histological examination of organs and population details, are needed to test this hypothesis.

PCV3 was less prevalent in this wild boar population than PCV2. It was detectable by qPCR in 25% of wild boar samples, which compared well to findings from Italy [65], where 30% of wild boars tested positive. The reported prevalence of PCV3 in two feral pig populations around Berlin (Germany) was 23% and 50%, respectively [15]. In our study, double infections of PCV2 and -3 occurred at a rate of 23%. This was considerably higher than the 12.5% and 7.5%, respectively, reported by Prinz et al. (2019) [15] for Germany. Interestingly, PCV3 was significantly more prevalent in adult animals, while viral loads between juvenile and adult animals were comparable, suggesting a higher susceptibility/likelihood of exposure of adult animals. Our findings support the theory that PCV3 is endemic globally [66]. However, whether PCV3 infection has a detrimental impact for wild animal health is still a matter of debate.

Despite their name, PCVs can also be found in other species than swine, such as ruminants, which Wang et al. (2018) [39] and Zhai et al. (2019) [41] showed for PCV2, and Franzo et al. (2019) [37] showed for PCV3. In our study, three red deer samples (4.7% of red deer) tested positive for PCV2 with relatively low viral loads of 1.46 × 10^6^, 1.35 × 10^7^, and 7.25 × 10^6^ GE/mL tissue and swab lysate. In contrast, PCV3 qPCRs of all ruminant samples produced negative results. The recently discovered PCV4 [67,68] was not included in this study, but should be considered in future epidemiological investigations.

PrV-specific nucleic acids could not be amplified from any porcine sample, implying a low virus prevalence and hence a low risk for transmission to the domestic pig population or dead-end hosts such as hunting dogs.

In both ruminant and wild boar samples, gammaherpesvirus infections seem to be common. PLHV specific nucleic acids were detected in 78 out of 95 (82%) wild boar samples, with PLHV-3 being the most prevalent herpesvirus in this study (69%), followed by PLHV-2 (46%) and PLHV-1 (11%). This is in contrast to findings from domestic swine from Germany, Italy, and Ireland, where PLHV-1 was most prevalent with 54% [49], 29% [50], and 74% [69], respectively. The prevalence of PLHV-2 ranged from 11–45% and of PLHV-3 from 4.5–48% [49,50,69]. In these studies, a co-infection of PLHV-1 and -3 was most frequently observed, followed by co-infections with PLHV-1 and -2 [50,69], or with all three PLHVs [49]. In the population presented here, no co-infections of PLHV-1 and -2 were observed, but co-infection with PLHV-2 and -3 was most frequently observed. Therefore, it should be further elucidated whether the dynamics of PLHV infections differ between domestic pigs and wild boar, and whether the geographical area and the organ sampled affects prevalence. Interestingly, the viral loads reported by Franzo [50] in domestic pigs were the highest for PLHV-2, while the viral loads in the presented study were highest for PLHV-3. PLHV-3 is genetically more distinct from PLHV-1 and -2, which were detected in feral pigs previously [48,49]. Franzo et al. (2021) [50], and McMahon et al. (2006) [69], did not observe any connections between gammaherpesvirus infections and disease in domestic pigs. From the data available in this study, there is no evidence that PLHVs are associated with either a lower body condition score or increased comorbidities at necropsy in the studied wild boar population, apart from a lower viral load of PLHV-3 when intestinal parasites were absent. A slight variation (not significant) was also observed for PHLV-1, with seemingly higher viral loads in adult animals. No statistically significant variation in PLHV virus loads was detectable between the samples in 2019 and 2020. This finding indicates a constant, low-level replication of PLHVs in this wild boar population.

Some gammaherpesviruses of small ruminants (OvHV-2, CpHV-2, and AlHV-1) are known to cause malignant catarrhal fever in other species [53,54,70,71], but none of these MCF associated viruses were detectable by pan-herpesvirus PCR in the wild ruminant samples.

However, in two red deer samples, sequences closest related to elk gammaherpesvirus were identified, which supports findings from China [57], postulating that this virus is adapted to other deer species besides elk. Sequences similar to fallow deer herpesvirus, which belong to the genus *Rhadinovirus*, were present in eight red deer and two fallow deer samples. Again, the clinical relevance of these viruses is unclear, but should not be neglected.

As a part of a monitoring program from 2007 to 2009, the Institute for Veterinary Investigations of the AGES (Austrian Agency for Health and Food Safety) tested 700 red deer samples as negative for the presence of BVDV via AG-ELISA and RT-qPCR [72]. One BVDV-1 antibody positive, 9-year-old male red deer was identified in a study published 2004 [60], in which 133 wild ruminants were tested. These surveillance data indicate that the prevalence of BVDV circulation in wildlife in Austria is low. In our study, no pestivirus-specific nucleic acids were detectable in any of the samples. Therefore, a low to absent circulation of pestiviruses in the sampled population can be assumed.

## 4. Materials and Methods

### 4.1. Animals and Samples

Samples were derived from animals shot during the annual driven hunts in autumn 2019 and 2020 in a private game enclosure in eastern Austria. The enclosure was approximately 12 km^2^ in size and fenced off by high stone walls, making it highly unlikely that the animals had direct contact with free-ranging animals from outside. It is estimated—based on the results of the yearly hunt and observations of the local hunters—that the population consisted of approximately 300 red deer and 450 wild boars. The population could be considered a closed game population as no animals had been restocked for ten years. Supplementary food was provided to the animals.

The samples consisted of organs from 64 red deer (*Cervus elaphus*), 5 fallow deer (*Dama dama*), 6 mouflon (*Ovis gmelini musimon*), and 95 wild boars (*Sus scrofa*). Estimated age, nutrition status, and species are shown in Supplementary Table S2. All animals were shot by hunters. Sampling and necropsy were performed on-site 1–3 h after death.

After the body cavities were opened, tissue samples were gained by aspiration of the lung and spleen tissue through a large-bore needle (14 G) into a sterile 10 mL disposable syringe. Additionally, tonsillar swabs were taken; samples were frozen at −80 °C until further processing.

### 4.2. Nucleic Acid Extraction and PCRs

Approximately 100 mg of spleen and lung samples were tissue lysed (Tissue lyser II, Qiagen, Germany) in 1 mL PBS and centrifuged at 16.000× *g* for 3 min. Tonsillar swabs were vortexed for 10 s in 1 mL sterile PBS. Equal parts of swab lysate and supernatant of the tissue lysate were pooled, and 200 µL were extracted employing the QIAamp 96 Virus QIAcube HT Kit using QIAcube HT (Qiagen, Hilden, Germany) according to the manufacturer’s instructions.

A PCV2 and PCV3 assay analyzing all 170 samples was carried out as a duplex qPCR. Only wild boar extracts were screened for PLHV-1, -2, and -3 by three individual qPCRs. A 10-fold dilution series of defined DNA plasmid standards were tested side by side with the samples for absolute quantification. The samples were considered positive if the qPCR demonstrated more than 10^5^ copies/mL (PCV2/PCV3) and 10^4^ copies/mL tissue and swab lysate (PLHV-1/PLHV-2/PLHV-3), respectively. All qPCRs were carried out in a Rotor-Gene Q 5-plex machine (Qiagen, Hilden, Germany).

For detection of herpesvirus-specific DNA and RNA of classical pestiviruses (BVDV and BDV) in ruminants and PrV specific DNA of wild boar samples, extracts were analyzed in pools of five. RT-PCR products were visualized using the capillary electrophoresis device QIAxcel Advanced System (Qiagen, Hilden, Germany). In the case of positive results, the respective pool was opened, and samples were analyzed individually.

Specific PCR products generated in the pan-PCRs were sanger sequenced (Eurofins Genomics, Ebersberg, Germany) and compared to published sequences with BLAST (NCBI, Bethesda, MD, USA).

Primer and probe sequences, as well as used PCR kits, are shown in Supplementary Table S3 [27,49,73,74,75,76].

### 4.3. Statistical Analysis

Statistical analysis was performed to assess the significance of results employing the Student’s *t*-test or—in the case of qualitative data—the Fisher’s exact test.

## 5. Conclusions

The presented data demonstrate a high prevalence of PLHVs and PCV2 in a closed wild boar population in Austria, and are the first quantitative data on PLHV-1 to -3 loads in wild boar. The studied population was free from important, notifiable pathogens such as PrV and BVDV, suggesting a low risk of spill-over into the domestic animal population.

## Figures and Tables

**Table 1:** Detection of herpesviruses in deer. The herpesvirus species was determined by sequence analysis of the final PCR product.

	2019	2020
fallow deer herpesvirus	3 (7.7%)	7 (19.4%)
elk gamma herpesvirus	1 (2.6%)	1 (2.8%)
no herpesvirus	35 (89.7%)	28 (77.8%)
sum	39	36

PrV (SuHV-1) infections in wild boar and pestivirus infections in wild ruminants were not detected by (RT-)PCR.

## Data Availability

Not applicable.

## Supplementary Materials

The following supporting information can be downloaded at https://www.mdpi.com/article/10.3390/pathogens11030305/s1. Table S1: All data used in this study. Table S2: Number of animals in the study, depending on sex and age, weight, and body score. Table S3: Primer, probes and kits employed in this study. Figure S1: Association of parasite burden/parasitic lesions with viral loads.
