# Supplementary material for: Porcine Circoviruses and Herpesviruses Are Prevalent in an Austrian Game Population
Source: Pathogens. 2022 Feb 28;11(3):305. doi: 10.3390/pathogens11030305 (PMC8953168; doi:10.3390/pathogens11030305)
Supplement: Supplementary file 1 [file pathogens-11-00305-s001.zip › pathogens-1526455-supplementary.pdf]

A

							PCV-2 qPCR	PCV-3 qPCR	Pan-Herpes PCR	Pan-Pesti PCR	lesion present = 1	lesion absent = 0			
year	sample identification	species	Sex	age	weight (kg)	body condition (0-4)	GE/ml Lysat final	GE/ml Lysat final	species		intestinal inflamma	lung lesions	liver lesions	lesions lymp	lesions splee
2019	H1	red deer	m	juv	68.3	3	neg	neg	Elk gamma HV	neg	1	1	1	0	0
	H2	red deer	m	juv	70	3	neg	neg		neg	0	0	0	0	0
	H3	fallow deer	w	ad	48	3	neg	neg		neg	0	0	0	0	0
	H4	red deer	w	ad	67.35	3	neg	neg	Fallow deer LHV	neg	0	0	1	0	1
	H5	red deer	w	ad	49.7	3	neg	neg	Fallow deer LHV	neg	1	0	1	1	0
	H6	red deer	w			3	neg	neg		neg	0	0	0	0	0
	H7	red deer	m	ad	132.8	3	neg	neg		neg	0	0	0	0	0
	H8	mufflon	m	juv	20	1	neg	neg		neg	1	0	1	1	0
	H9	red deer	w	juv	55	3	neg	neg		neg	0	0	0	0	0
	H10	red deer	w	juv	58	3	neg	neg		neg	0	0	1	0	0
	H11	red deer	w			3	neg	neg		neg	1	0	0	0	0
	H12	fallow deer	w	ad	40	3	neg	neg		neg	0	0	0	0	0
	H13	red deer	m	ad	110	3	neg	neg		neg	0	0	1	0	0
	H14	red deer	m	ad	145	3	neg	neg		neg	0	0	0	1	0
	H15	red deer	m	ad	115	3	neg	neg		neg	0	1	0	0	0
	H16	red deer	m	ad	100	3	neg	neg		neg	1	0	0	0	0
	H17	red deer	w	juv	60	3	neg	neg		neg	0	0	0	0	0
	H18	red deer	w	juv	65	3	neg	neg		neg	1	1	0	0	0
	H19	red deer	w	juv	58	3	neg	neg		neg	0	0	0	0	0
	H20	red deer	m	juv	85	3	neg	neg		neg	0	0	0	1	0
	H21	red deer	w	ad	82	3	neg	neg		neg	0	0	0	1	0
	H22	red deer	w	ad	95	3	neg	neg		neg	1	0	1	0	0
	H23	red deer	w	ad	75	3	neg	neg		neg	0	0	0	0	0
	H24	red deer	w	juv	35	1	neg	neg		neg	1	0	0	0	0
	H25	red deer	w	juv	52.7	3	neg	neg		neg	0	0	0	1	0
	H26	red deer	m	ad	110	3	neg	neg		neg	1	0	0	1	0
	H27	red deer	m	ad	120	3	neg	neg		neg	0	0	0	1	0
	H28	red deer	w	juv	45	3	neg	neg		neg	0	0	0	0	0
	H29	red deer	m	ad	110	3	neg	neg		neg	0	0	0	0	0
	H30	red deer	w	juv	65	3	neg	neg		neg	0	0	0	0	0
	H31	red deer	w	ad	70	3	neg	neg		neg	0	0	0	0	0
	H32	fallow deer	w			3	neg	neg		neg	nd	nd	nd	nd	nd
	H33	red deer	w	ad		3	neg	neg		neg	nd	nd	nd	nd	nd
	H34	red deer	w	juv	60	3	neg	neg		neg	1	0	1	1	0
	H35	red deer	w	juv	55	3	neg	neg		neg	1	0	0	0	0
	H36	fallow deer	w	juv	45	3	neg	neg	Fallow deer LHV	neg	0	0	0	1	0
	H37	red deer	w	ad		3	neg	neg		neg	nd	nd	nd	nd	nd
	H38	red deer	w	juv	50	3	neg	neg		neg	0	0	0	1	0
	H39	red deer	m	ad	138	3	1.46E+06	neg		neg	0	0	0	0	0
2020	H1	red deer	m	juv	53.5	2	neg	neg		neg	1	0	0	1	0
	H2	red deer	m	ad	76.5	1	neg	neg		neg	0	1	0	1	0
	H3	mufflon	w	ad	32	2	neg	neg		neg	0	1	1	0	0
	H4	red deer	w	ad	58	2	neg	neg	Fallow deer LHV	neg	1	1	0	1	0
	H5	red deer	m	juv	66	2	neg	neg		neg	0	0	0	1	0
	H6	red deer	m	juv	75	2	neg	neg	Fallow deer LHV	neg	1	0	0	1	0
	H7	mufflon	w	ad	21.8	2	neg	neg		neg	0	0	0	0	0
	H8	red deer	m	ad	92	2.5	neg	neg		neg	1	0	0	1	0
	H9	red deer	m	juv	67	2	neg	neg		neg	0	0	0	1	0
	H10	red deer	m	juv	62	1	neg	neg	Fallow deer LHV	neg	0	0	0	1	0
	H11	red deer	w	juv	62	2.5	neg	neg		neg	0	0	0	0	0
	H12	red deer	w	juv	66	2	neg	neg		neg	0	0	0	0	0
	H13	red deer	m	ad	113	2	neg	neg		neg	0	0	0	0	0
	H14	red deer	w	juv	62.5	2	neg	neg	Fallow deer LHV	neg	0	1	0	0	0
	H15	red deer	w	ad	105	2	neg	neg		neg	0	0	0	0	0
	H16	red deer	m	ad	94	2	neg	neg		neg	0	1	0	1	0
	H17	red deer	m	juv	72	2.5	neg	neg		neg	1	0	0	1	0
	H18	red deer	m	juv	58	1	neg	neg		neg	nd	nd	nd	nd	nd
	H19	red deer	m	ad	145	2.5	neg	neg		neg	0	0	0	0	0
	H20	red deer	w	ad	110	2.5	neg	neg		neg	0	0	1	0	0
	H21	red deer	m	juv	62	2	neg	neg	Fallow deer LHV	neg	0	0	0	1	0
	H22	red deer	w	juv	69	2	neg	neg	Elk gamma HV	neg	1	0	1	1	0
	H23	red deer	m	juv	63	2	neg	neg		neg	0	0	0	0	0
	H24	fallow deer	w	ad	47	2	neg	neg	Fallow deer LHV	neg	0	0	0	0	0
	H25	red deer	w	ad	100	2	neg	neg		neg	0	0	0	1	0
	H26	red deer	m	juv	61.5	1	neg	neg	Fallow deer LHV	neg	0	0	0	0	0
	H27	red deer	w	juv	73	2	neg	neg		neg	0	1	0	1	0
	H28	mufflon	w	ad	32	2	neg	neg		neg	0	1	1	0	0
	H29	red deer	m	juv	66	1	neg	neg		neg	0	1	1	0	0
	H30	mufflon	w	ad	31	2	neg	neg		neg	1	0	1	1	0
	H31	red deer	w	juv	74	2	neg	neg		neg	0	0	0	0	0
	H32	red deer	w	juv	55	1	neg	neg		neg	0	0	0	1	0
	H33	mufflon	w	ad	23	2	neg	neg		neg	1	1	1	0	0
	H34	red deer	w	juv	53	1	neg	neg		neg	0	0	0	0	0
	H35	red deer	w	juv	71	2	1.35E+07	neg		neg	1	0	0	0	0
	H36	red deer	m	juv	57.5	1	7.25E+06	neg		neg	1	0	0	1	0

Supplementary table S1: Data generated in this study. (A) ruminants. (B) wild boar.

B

							PCV-2 qPCR	PCV-3 qPCR	qPLHV-1	qPLHV-2	qPLHV-3	lesion present = 1	lesion absent = 0	
year	sample identification	species	Sex	age	weight (kg)	body condition (0-4)	GE/ml Lysat final	GE/ml Lysat final	GE/ml Lysat final	GE/ml Lysat final	GE/ml Lysat final	intestinal parasites	milk spots	lung worms
2019	S1	wild boar	w	ad	60	3	neg	neg	neg	neg	neg	1	1	1
	S2	wild boar	m	ad	90	3	3.08E+05	neg	neg	neg	neg	1	0	0
	S3	wild boar	w	ad	60	3	neg	neg	neg	neg	1.78E+05	1	0	0
	S4	wild boar	m	ad	65.5	3	1.91E+07	9.45E+07	4.01E+05	neg	6.75E+05	1	0	1
	S5	wild boar	m	ad	95.5	3	1.23E+06	neg	neg	4.97E+04	7.15E+05	1	0	0
	S6	wild boar	w	ad	60	3	5.10E+05	neg	neg	neg	neg	1	0	0
	S7	wild boar	m	juv	28.8	2	neg	neg	neg	neg	1.38E+06	1	0	0
	S8	wild boar	m	ad	116.5	3.5	neg	neg	neg	neg	neg	1	0	0
	S9	wild boar	m	ad	82.5	3.5	2.79E+07	4.37E+06	neg	1.24E+05	2.72E+05	0	0	0
	S10	wild boar	m	ad	87.5	3	neg	neg	neg	neg	neg	0	0	1
	S11	wild boar	m	ad	91.5	3	1.25E+05	neg	neg	2.91E+04	1.23E+04	0	0	0
	S12	wild boar	m	ad	63	3	2.89E+07	5.50E+05	neg	neg	neg	1	0	1
	S13	wild boar	m	ad	89	3	neg	neg	neg	neg	4.68E+05	1	0	0
	S14	wild boar	m	ad	186	3.5	neg	neg	neg	7.45E+04	4.48E+04	0	0	0
	S15	wild boar	w	ad	61.5	3	7.85E+05	neg	neg	neg	1.75E+06	1	0	0
	S16	wild boar	w	juv	18	2	5.28E+07	neg	neg	2.38E+05	7.05E+04	1	0	1
	S17	wild boar	w	juv	18	3	1.66E+08	neg	neg	1.67E+04	7.35E+05	0	0	1
	S18	wild boar	w	ad	58	3	9.15E+07	neg	neg	2.33E+04	1.31E+04	1	0	0
	S19	wild boar	m	ad	54	3	1.84E+07	1.82E+06	neg	neg	neg	1	0	0
	S20	wild boar	m	juv	22	2	3.57E+08	neg	neg	neg	neg	1	0	0
	S21	wild boar	w	juv	31.4	3	4.52E+05	neg	neg	neg	neg	1	0	1
	S22	wild boar	m	ad	63	3	1.90E+05	neg	neg	neg	neg	1	0	1
	S23	wild boar	m	ad	50	3	9.93E+06	2.68E+05	neg	neg	3.00E+05	1	0	0
	S24	wild boar	w	juv	33.4	2	neg	neg	neg	neg	4.46E+04	1	0	1
	S25	wild boar	m	juv	39	3	1.24E+05	2.44E+05	neg	neg	6.05E+05	1	1	1
	S26	wild boar	m	ad	45.2	3	8.28E+06	neg	1.77E+04	neg	neg	1	1	0
	S27	wild boar	w	ad	56	2	5.10E+08	1.87E+07	neg	neg	1.08E+04	1	0	1
	S28	wild boar	m	ad	109	3	4.10E+05	1.06E+05	neg	1.03E+04	2.90E+05	1	0	0
	S29	wild boar	w	ad	50	2	neg	neg	neg	2.71E+04	neg	0	0	0
	S30	wild boar	m	ad	66.5	2	neg	neg	neg	neg	2.13E+04	1	0	1
	S31	wild boar	m	juv	17.8	1	2.25E+05	neg	8.10E+04	neg	8.30E+05	1	0	0
	S32	wild boar	w	ad	59	3	neg	neg	neg	1.47E+04	neg	1	0	0
	S33	wild boar	w	ad	55.5	3	5.95E+05	neg	neg	1.26E+04	neg	1	1	1
	S34	wild boar	w	juv	28.6	2	8.75E+08	neg	neg	neg	neg	1	1	0
	S35	wild boar	w	ad	49	3	1.65E+06	neg	neg	neg	1.26E+05	1	0	0
	S36	wild boar	w	ad	65.9	3	8.70E+05	1.93E+05	neg	neg	6.40E+04	1	0	0
	S37	wild boar	w	ad	69	3	3.20E+05	neg	neg	1.38E+04	neg	1	0	1
	S38	wild boar	m	ad	46	2	neg	neg	neg	neg	1.31E+06	1	0	0
	S39	wild boar		ad	49.5	2	2.48E+05	neg	neg	2.63E+04	6.25E+05	1	0	1
	S40	wild boar	m	ad	98	2	1.85E+05	neg	neg	neg	neg	1	0	1
	S41	wild boar	w	ad	56	3	3.61E+05	neg	neg	neg	2.26E+05	1	1	1
	S42	wild boar	m	ad	102.5	3.5	4.95E+05	neg	neg	1.13E+05	1.98E+04	1	0	0
	S43	wild boar	m	juv	24.8	3	3.99E+05	neg	neg	neg	6.75E+05	0	0	0
	S44	wild boar		juv	26.4	3	1.91E+05	neg	neg	neg	1.51E+05	0	0	0
	S45	wild boar	m	ad	62.5	3	neg	neg	neg	neg	neg	1	1	1
	S46	wild boar	m	juv	29.3	3	1.12E+05	neg	neg	2.61E+04	8.45E+04	1	1	1
	S47	wild boar	m	juv	22.5	2	8.13E+07	5.26E+05	neg	neg	1.25E+06	1	0	0
	S48	wild boar	m	ad	94.5	2	neg	neg	neg	neg	1.05E+05	0	1	0
	S49	wild boar	w	juv	28	3	neg	neg	neg	neg	1.68E+04	1	0	1
	S50	wild boar	m	juv	20	2	neg	neg	neg	3.44E+04	5.10E+06	1	0	1
2020	WS2	wild boar	m	juv	33	2	1.05E+09	neg	neg	neg	neg	1	0	0
	WS3	wild boar	m	ad	109	2	neg	neg	neg	7.00E+04	6.20E+04	1	0	1
	WS4	wild boar	m	ad	83	2	neg	7.70E+05	neg	2.10E+04	neg	1	0	0
	WS5	wild boar	w	juv	34	2	neg	neg	neg	neg	neg	1	0	0
	WS6	wild boar	w	juv	27	2	5.40E+09	neg	neg	5.50E+04	2.33E+05	1	0	0
	WS7	wild boar	w	juv	20	1.5	neg	neg	neg	neg	1.07E+05	1	0	0
	WS8	wild boar	m	ad	85	2	neg	neg	neg	1.39E+05	2.17E+05	1	0	1
	WS9	wild boar	w	ad	60	2	7.80E+05	1.59E+07	8.20E+05	1.92E+04	1.21E+06	1	0	0
	WS10	wild boar	n	juv	16	2	neg	neg	1.50E+05	1.07E+04	2.27E+05	1	1	1
	WS11	wild boar	w	juv	21	2	neg	neg	neg	1.82E+04	1.62E+06	1	1	1
	WS12	wild boar	m	ad	71		3.17E+05	5.20E+05	neg	1.84E+04	7.25E+04	1	0	1
	WS13	wild boar	w	juv	26	1.5	5.35E+09	neg	neg	3.44E+04	7.10E+05	1	0	0
	WS14	wild boar	w	juv	28	1.5	8.40E+08	neg	7.05E+04	neg	6.75E+05	1	0	0
	WS15	wild boar	m	ad	106	2	9.50E+06	1.66E+05	neg	2.60E+05	3.11E+05	1	1	0
	WS16	wild boar	m	ad	95		1.11E+06	6.20E+05	7.45E+05	neg	neg	0	0	1
	WS17	wild boar	m	ad	115	3	5.90E+05	4.67E+05	neg	neg	2.30E+04	0	0	0
	WS18	wild boar	m	juv	40	2	5.75E+06	2.49E+07	neg	5.15E+04	3.48E+04	1	0	0
	WS19	wild boar	m	juv	35	1.5	2.59E+10	neg	neg	2.21E+05	3.50E+05	1	1	0
	WS20	wild boar	w	juv	28	1.5	1.50E+10	neg	neg	neg	neg	1	0	1
	WS21	wild boar	m	juv	48	2	7.20E+05	neg	neg	6.95E+04	8.20E+04	1	0	0
	WS22	wild boar	m	juv	35	2	2.99E+06	neg	neg	neg	2.84E+05	1	1	1
	WS23	wild boar	m	juv	25	2	4.79E+09	neg	4.44E+04	1.75E+04	2.75E+05	1	0	0
	WS24	wild boar	m	ad	85	2.5	1.02E+06	6.85E+06	neg	8.15E+04	6.65E+04	1	1	1
	WS25	wild boar	m	juv	18	2	4.18E+05	neg	neg	2.13E+05	neg	0	0	1
	WS26	wild boar	w	juv	28	2	neg	2.46E+05	neg	7.75E+05	3.25E+05	1	0	0
	WS27	wild boar	w	juv	29	2	2.09E+05	neg	neg	1.82E+04	6.45E+06	1	1	1
	WS28	wild boar	m	ad	115	3	3.33E+05	6.30E+05	neg	1.03E+05	2.61E+05	1	0	1
	WS29	wild boar	w	juv	45	2	2.30E+11	neg	neg	3.31E+04	2.20E+06	1	0	0
	WS30	wild boar	w	ad	54	2	5.10E+05	1.38E+07	3.96E+06	neg	3.22E+06	1	0	0
	WS31	wild boar	m	juv	23	2	1.12E+07	neg	neg	2.16E+05	neg	1	0	1
	WS32	wild boar	w	juv	27	2	5.75E+09	neg	neg	neg	6.45E+04	1	1	1
	WS33	wild boar	m	ad	49	2	1.39E+07	1.81E+05	neg	neg	2.89E+04	1	1	1
	WS34	wild boar	w	juv	25	2	4.70E+10	neg	neg	neg	9.95E+05	1	0	0
	WS35	wild boar	m	juv	36	2	1.67E+06	neg	neg	4.55E+04	3.38E+05	1	0	1
	WS36	wild boar	m	juv	25	2	2.85E+11	neg	neg	6.30E+04	3.23E+04	1	1	1
	WS37	wild boar	m	juv	26	2	6.85E+05	neg	neg	neg	neg	1	1	0
	WS38	wild boar	m	juv	24	2	1.04E+07	neg	neg	neg	8.65E+04	0	0	1
	WS39	wild boar	m	ad	46	2	3.20E+09	4.49E+07	neg	neg	2.06E+04	1	0	1
	WS40	wild boar	w	juv	39	2	2.32E+07	9.05E+07	neg	neg	3.45E+04	1	0	0
	WS41	wild boar	w	juv	36	2	1.13E+06	neg	neg	1.77E+04	neg	0	0	1
	WS42	wild boar	m	juv	39	2	5.30E+05	neg	neg	1.55E+05	neg	1	1	1
	WS43	wild boar	w	juv	33	2	1.88E+11	neg	neg	1.38E+04	2.07E+04	1	0	0
	WS44	wild boar	w	ad	65	2	9.85E+07	neg	1.16E+05	neg	neg	1	0	1
	WS45	wild boar	w	juv	29	2	7.55E+07	neg	neg	3.51E+05	5.60E+04	1	1	1
	WS46	wild boar	w	juv	37	2	6.10E+07	neg	neg	2.06E+04	1.48E+06	1	0	0

Supplementary table S1 continued: Data generated in this study. (A) ruminants. (B) wild boar.

			weight (kg)		body score (0-4)	
		N	average	STDEV	average	STDEV
**wild boar**	female ad	16	58.7	5.4	2.7	0.5
	female juv	23	29.1	6.6	2.0	0.4
	male ad	32	84.9	28.9	2.7	0.6
	male juv	21	29.1	8.2	2.1	0.5
**red deer**	female ad	12	81.2	20.6	2.7	0.5
	female juv	21	59.2	9.5	2.4	0.7
	male ad	14	114.4	20.4	2.6	0.6
	male juv	15	65.8	7.8	1.9	0.8
**fallow deer**	female ad	3	45.0	4.4	2.7	0.6
	female juv	1	45.0	-	3.0	-
	male ad	0	-	-	-	-
	male juv	0	-	-	-	-
**mouflon**	female ad	5	28.0	5.1	2.0	0.0
	female juv	0	-	-	-	-
	male ad	0	-	-	-	-
	male juv	1	20.0	-	1.0	-

Supplementary table S2: Number of animals in the study, depending on sex and age, weight and body score.

Virus	Primer/Probe sequences	Reference	PCR Kit
**PCV2**	qPCV2-F: 5'-GAGTCTGGTGACCGTTGCA-3'<br>qPCV2-R: 5'-YCCCGCTCACTTTCAAAAGTTC-3'<br>qPCV2-Probe: FAM-5'-CCCTGTAACGTTTGTCAGAAATTTCCGCG-3'-BHQ1	Hoffmann, 2016	Luna® Universal Probe qPCR Master Mix (NEB)
**PCV3**	qPCV3-F: 5'-AGTGCTCCCCATTGAACG-3'<br>qPCV3-R: 5'-ACACAGCCGTTACTTCAC-3'<br>qPCV3-Probe: Cy5-5'-ACCCCATGGCTCAACACATATGACC -3'-BHQ2	Palinski et al., 2007	
**PLHV1**	qPLHV1-F: 5'-CTCACCTCCAAATACAGCGA-3'<br>qPLHV1-R: 5'-GCTTGAATCGTGTGTTCCATA-3'<br>qPLHV1-Probe: FAM-5'-CTGGTCTACTGAATCGCCGCTAACAG-3'-BHQ-1	Chmielewitz et al., 2003	
**PLHV2**	qPLHV2-F: 5'-GTCACCTGCAAATACACAGG-3'<br>qPLHV2-R): 5'-GGCTTGAATCGTATGTTCCATAT-3'<br>qPLHV2-Probe: FAM-5'-CTGGTCTACTGAAGCGCTGCCAATAG-3'-BHQ-1		
**PLHV3**	qPLHV3-F: 5'-AAGGACCCCAAAGAGGAAA-3'<br>qPLHV3-R: 5'-CTGAGGCACTGCATACTCTGT-3'<br>qPLHV3-Probe: FAM-5'-TCAATTTTATGGTTCACCTTCTACCTTTCCT-3'-BHQ-1		
**Herpes-**<br>**viruses**<br>**(pan-PCR)**	HerpesCons-F1 5'-GAYTTYGCNAGYYTNTAYCC-3'<br>HerpesCons-F2 5'-TCCTGGACAAGCAGCARNYSGCNMTNAA-3'<br>HerpesCons-R 5'-GTCTTGCTCACCAGNTCNACNCCYTT-3'<br>HerpesCons-nF 5'-TGTAACTCGGTGTAYGGNTTYACNGGNGT-3'<br>HerpesCons-nR 5'-CACAGAGTCCGTRTCNCCRTADAT-3'	Van Devanter et al., 1996	QIAGEN Fast Cycling PCR Kit (Qiagen, Germany)
**PrV**	ADV-F: 5'-ATGGCCATCTCGCGGTGC-3'<br>ADV-R: 5'-ACTCGCGGTCCTCCAGCA-3'	Mengerling et al., 1992	
**Pesti-**<br>**viruses**<br>**(pan-PCR)**	PanPesti-1F: 5`-CATGCCCTCAGTAGGACTAGC-3`<br>PanPesti-nF: 5`-CTCGAGATGCCACGTGGACGAGG-3`<br>PanPesti-1R/nR: 5`-CTCCATGTGCCATGTAGAGCAGAG-3´	Yesilbag et al., 2008<br>(mod. nach Lamp B.)	One Taq® One-Step RT-PCR Kit (NEB) und QIAGEN Fast Cycling PCR Kit (Qiagen, Germany)

Supplementary table S3: Primer, probes and kits employed in this study.

	milk spots +	milk spots -	intestinal parasites +	intestinal parasites -	lungworms +	lungworms -	milk spots +	milk spots -	intestinal parasites +	intestinal parasites -	lungworms +	lungworms -	milk spots +	milk spots -	intestinal parasites +	intestinal parasites -	lungworms +	lungworms -	milk spots +	milk spots -	intestinal parasites +	intestinal parasites -	lungworms +	lungworms -	milk spots +	milk spots -	intestinal parasites +	intestinal parasites -	lungworms +	lungworms -
**N**	**17**	**54**	**61**	**10**	**33**	**38**	**4**	**20**	**21**	**3**	**10**	**14**	**2**	**8**	**9**	**1**	**4**	**6**	**11**	**33**	**37**	**7**	**22**	**22**	**15**	**51**	**57**	**9**	**26**	**31**
juv	11	26	31	6	17	20	1	4	5	0	1	4	1	3	4	0	1	3	8	16	21	3	14	10	10	25	31	4	14	19
ad	6	28	30	4	16	18	3	16	16	3	9	10	1	5	5	1	3	3	3	17	16	4	8	12	5	26	26	5	12	12

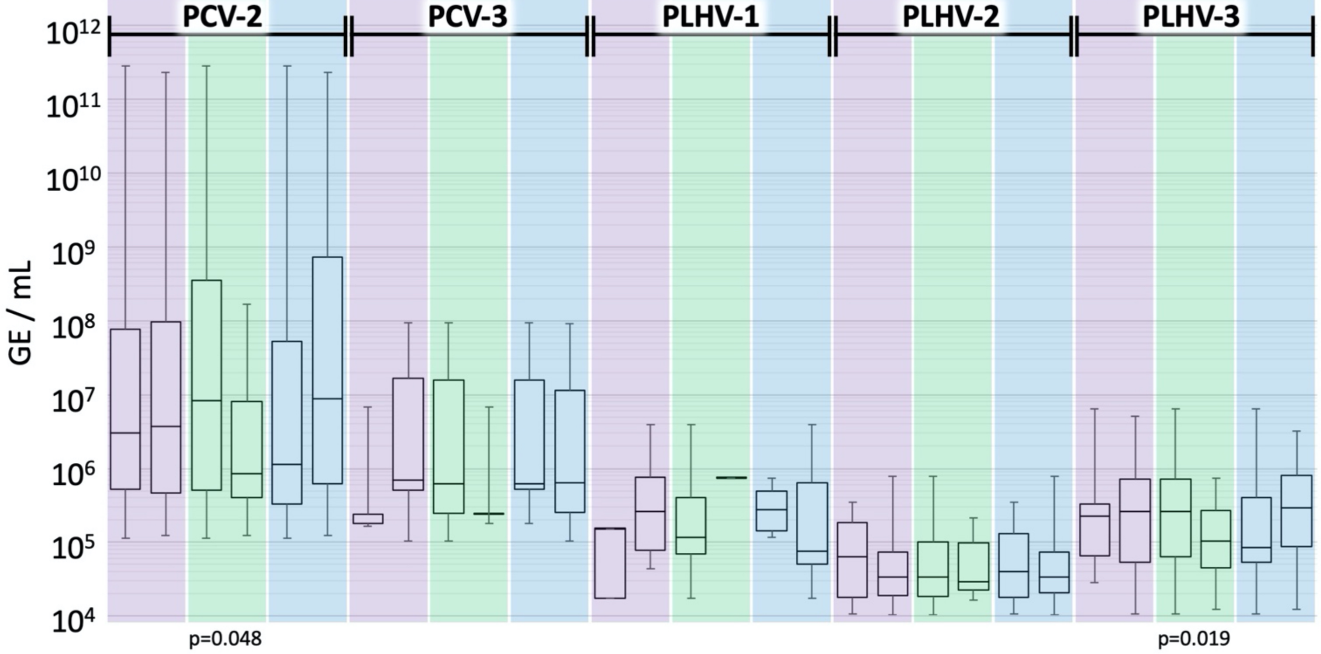

Supplementary figure S1: Association of parasite burden / parasitic lesions with viral loads. Indicated are the total number of animals in each group (N), the age group (juv [juvenile] and ad [adult]) and whether lesions were present (+) or not (-). Lesions analysed were milk spots (purple), intestinal parasites (light green) and lung worms (blue). Statistical analysis was performed when at least ten animals each were present in the group with and without lesions. P values are indicated when lower than 0.05.
